# Effect of parity on metabolic and oxidative stress profiles in Holstein dairy cows

**DOI:** 10.14202/vetworld.2020.2780-2786

**Published:** 2020-12-26

**Authors:** Shimaa G. Yehia, Eman S. Ramadan, Eissa A. Megahed, Noha Y. Salem

**Affiliations:** 1 Department of Internal Medicine and Infectious Diseases, Faculty of Veterinary Medicine, Cairo University, Giza, Egypt; 2 Veterinary Medicine Directorate - Giza, El Haram, Giza, Egypt

**Keywords:** haptoglobin, heifers, multiparous, non-esterified fatty acids, oxidative stress, transition

## Abstract

**Background and Aim:**

Pregnancy and lactation have an impact on health status of animals and constitute burden on body metabolites and the oxidant-antioxidant equilibrium. This study is aimed at evaluating metabolic and oxidative stress patterns and parity impacts in both primiparous and multiparous dairy cows.

**Materials and Methods:**

Twenty-seven primiparous and multiparous. Holstein cows were enrolled and categorized into four groups according to their physiologic status: Primiparous peripartum heifer (n=5), primiparous postpartum cow (n=9), multiparous peripartum cows (n=5), and multiparous postpartum cows (n=8). Blood sample was taken from each animal – peripartum groups at 3 weeks prepartum and postpartum groups at 3 weeks post-parturition – for complete blood picture, glucose, cholesterol, triglyceride, total protein, albumin, non-esterified fatty acids (NEFA), malondialdehyde (MDA), total antioxidant capacity, and haptoglobin estimation.

**Results:**

Postpartum primiparous cows showed significant decrease in glucose, total protein, and albumin while showing significant increase in MDA, NEFA, and globulin; on the other hand, multiparous postpartum cows showed significant decrease in glucose, total protein, and albumin, associated with significant increase in cholesterol and MDA when compared with prepartum PP and MP cows, respectively. Postpartum multiparous cows significantly showed reduction in NEFA when compared to primiparous postpartum cows. Hematologic profiles of postpartum primiparous and multiparous cows showed significant decrease in red blood cells and packed cell volume, significant increase in lymphocytes when compared with prepartum cows.

**Conclusion:**

Metabolic and oxidative abnormalities exist in both primiparous and multiparous cows during the transition phase, however postpartum primiparous cows show higher susceptibility to negative energy balance impacts. Oxidant/antioxidant imbalance occurred in both the primiparous and multiparous postpartum cows, highlighting the importance of oxidative stress profiles in the assessment of metabolic health status during transition.

## Introduction

As previously defined, transition period is the time frame from 3 weeks before calving to 3 weeks after calving [1]. Transitioning cows then undergo numerous physiological and biochemical alterations, which act as stressors, and in turn, the cows become more vulnerable to several metabolic and infectious conditions [2,3]. During the period of early lactation and colostrum production, a cow’s demand for energy and nutritional diet will significantly improve. This period is also crucial since it is usually associated with a reduction in food intake, and consequently, negative energy balance (NEB) is expected and mobilization of body fat in the form of non-esterified fatty acids (NEFA) will ensue [4].

Clinicopathological evaluation is one means of assessing dairy cow health status at the individual and herd level [5]. Several factors such as parity, stage of lactation, and season of production have substantial effects on milk production and blood parameters in clinically normal lactating cows [6]. One study found that the periparturient phase associated with liver inflammatory response is linked to mobilization of fats [4], leading to an increased lipid mobilization during the transition period due to the generation of reactive oxygen species (ROS) caused by NEB [7]. Although ROS are normal products of metabolic processes and are not generally harmful, it may lead to oxidative stress when found in greater amounts, or when produced at faster rates to an extent that antioxidant mechanisms can no longer mitigate them [8]. Haptoglobin (HP) is an acute-phase protein synthesized in the liver and could be used as a predictor of non-specific immune response caused by several inflammatory reactions [9]. NEB can lead to an increase in concentrations of HP around parturition [10]. Moreover, early lactation phase can cause tremendous loss in body fats in high producing dairy cows [1].

This study is aimed at evaluating the effects of parity on hematobiochemical and oxidative stress biomarkers as well as studying the metabolic and oxidative stress patterns in both primiparous and multiparous Holstein dairy cows during the transition phase.

## Materials and Methods

### Ethical approval

The research procedures conducted in this study were approved by the Institutional Animal Care and Use Committee (Approval code: Vet CU16072020178), Faculty of Veterinary Medicine, Cairo University, Egypt.

### Study period and location

The study was conducted between September 2019 and December 2019. The samples were collected from Dairy farm in Fayoum Governate, Egypt and the laboratory investigations were conducted at Medicine and Infectious Diseases Department Laboratory, Faculty of Veterinary Medicine, Cairo University.

### Animals

**Figure-1:**
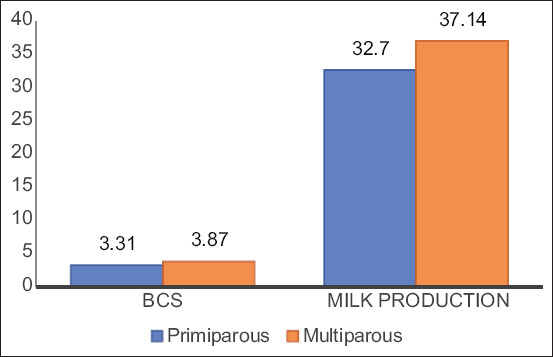
Body condition score and milk yield in primiparous and multiparous cows

We enrolled 27 Holstein cows (weight: 450-700 kg and age: 2-6 years) into the study. Milk production and body condition scores (BCS) are depicted in Figure-1. Studied animals were categorized into four groups according to physiological status: (1) Primiparous peripartum group (n=5), (2) multiparous peripartum group (n=5): One sample was withdrawn from each animal 3 weeks preparturition, (3) primiparous postpartum group (n=9), and (4) multiparous postpartum group (n=8): One sample was withdrawn from each animal at 3^rd^ week postpartum.

Inclusion criteria included the following: Cows/heifers with no prior clinical health issue and normal parturition without any adverse events and a BCS of 3 or more. Concerning multiparous cows, lactation period should be more than 200 days with an average milk production of more than 33 L/day.

### Samples and laboratory investigations

Blood samples from each cow were collected on two tubes through tail vein. The first tube containing ethylenediaminetetraacetic acid was used for complete blood count using automated veterinary hematology analyzer. The second was a plain tube used for serum separation. Serum was then used to estimate glucose, cholesterol, triglyceride, total protein, and albumin (Spectrum Diagnostics, Egypt). NEFA was analyzed in all the collected serum samples (Randox, Cat no. FA115, UK). Malondialdehyde (MDA) and total antioxidant capacity (TAC) were estimated in the collected serum using dedicated test kits (Bio Diagnostic, Egypt). HP testing was done on serum samples (HP immunoturbidimetry) [11].

### Statistical analysis

All quantitative data of hematology and metabolic and oxidative stress markers were presented as mean± standard error. The comparison was made using SPSS Statistics program version 16.0 (SPSS Inc. Released 2007. SPSS for Windows, Chicago) (independent samples t-test). p≤0.05 was considered significant.

## Results

### Serum biochemistry and oxidative stress markers

When compared to the prepartum primiparous group, the postpartum primiparous cows showed a significant decrease in glucose, total protein, and albumin yet a significant increase in MDA, NEFA, and globulin (Table-1). Moreover, the multiparous postpartum group showed a significant decrease in glucose, total protein, and albumin, associated with a significant increase in cholesterol and MDA when compared with the prepartum multiparous group (Table-2). The findings also showed that the postpartum multiparous group showed a significant decrease in NEFA associated, with a non-significant increase in cholesterol, total protein, TAC, and MDA, compared to the primiparous postpartum group, in which the latter showing a non-significant increase in glucose (Table-3).

**Table-1:** Metabolic, oxidative stress, and hematologic profile in primiparous cows before and after parturition.

Parameter/unit	Peripartum primiparous group (n=5)	Postpartum primiparous group (n=9)
Glucose (mmol/L)	2.85±0.13	2.11±0.25*
NEFA (mmol/L)	0.37±0.03	0.91±0.12*
Cholesterol (mmol/L)	2.62±0.25	2.82±0.34
Triglyceride (mmol/L)	0.43±0.06	0.44±0.07
Total Protein (g/L)	69.3±1.4	59.0±3.5*
Albumin (g/L)	34.8±1.2	11.9±0.3*
Globulin (g/L)	32.5±1.3	43.9±1.0*
Haptoglobin (Mmol/L)	2.2±0.5	3.3±0.2
TAC (mM/L)	0.68±0.16	1.08±0.17
MDA (nmol/ml)	1.54±0.36	2.29±0.39*
RBCs (10^12^/L)	8.53±0.44	6.72±0.16*
PCV (L/L)	0.33±0.01	0.28 ±0.02*
HB (g/L)	108.4±4.2	102.2±6.2
WBCs (10^9^/L)	13.20±0.39	16.48±1.43
Lymphocytes (10^9^/L)	6.74±0.26	10.51±1.62*
Neutrophils (10^9^/L)	5.30±0.27	5.05±0.63
Platelets (10^9^/L)	252.50±18.80	230.50±23.80

Data were presented as mean±standard error. p≤0.05 was considered significant. NEFA=Non-esterified fatty acids, TAC=Total antioxidant capacity, MDA=Malondialdehyde, RBCs=Red blood cells, PCV=Packed cell volume, HB=Hemoglobin, WBCs=White blood cells

**Table-2:** Metabolic, oxidative stress, and hematologic profile in multiparous cows before and after parturition.

Parameter/unit	Peri-partum multiparous group (n=5)	Postpartum multiparous group (n=8)
Glucose (mmol/L)	2.75±0.04	1.81±0.23*
NEFA (mmol/L)	0.27±0.02	0.34±0.05
Cholesterol (mmol/L)	3.05±0.05	3.59±0.21*
Triglyceride (mmol/L)	0.56±0.02	0.43±0.07
Total Protein (g/L)	78.4±5.1	64.2±0.4*
Albumin (g/L)	35.8±0.4	11.5±0.4*
Globulin (g/L)	42.5±5.1	48.8±4.0
Haptoglobin (mmol/L)	3.9±1.0	3.1±0.03
TAC (mM/L)	1.01±0.26	1.26±0.003
MDA (nmol/ml)	1.08±0.26	2.52±0.31*
RBCs (10^12^/L)	8.66±0.30	6.43±0.20*
PCV (L/L)	0.34±0.004	0.28±0.01*
HB (g/L)	113.8±2.3	100.4±5.6
WBCs (10^9^/L)	12.23±0.45	14.53±1.09
Lymphocytes (10^9^/L)	5.49±0.88	8.80±0.65*
Neutrophils (10^9^/L)	5.81±0.72	4.40±0.18
Platelets (10^9^/L)	242.20±45.33	229.20±16.67

Data were presented as mean±standard error. p≤0.05 was considered significant. NEFA=Non-esterified fatty acids, TAC=Total antioxidant capacity, MDA=Malondialdehyde, RBCs=Red blood cells, PCV=Packed cell volume, HB=Hemoglobin, WBCs=White blood cells

**Table-3:** Metabolic, oxidative stress, and hematologic profile in postpartum primiparous and multiparous cows.

Parameter/unit	Postpartum primiparous group (n=9)	Postpartum multiparous group (n=8)
Glucose (mmol/L)	2.11±0.25	1.81±0.23
NEFA (mmol/L)	0.91±0.12	0.34±0.06*
Cholesterol (mmol/L)	2.82±0.34	3.59±0.21
Triglyceride (mmol/L)	0.44±0.07	0.43±0.07
Total Protein (g/L)	59.0±3.5	64.2±0.4
Albumin (g/L)	11.9±0.3	11.5±0.4
Globulin (g/L)	43.9±1.0	48.8±4.0
Haptoglobin (mmol/L)	3.3±0.2	3.1±0.03
TAC (mM/L)	1.075±0.17	1.26±0.003
MDA (nmol/ml)	2.29±0.39	2.52 ±0.31
RBCs (10^12^/L)	6.72±0.16	6.43±0.20
PCV (L/L)	0.28 ±0.02	0.28±0.01
HB (g/L)	102.2±6.2	100.4±5.6
WBCs (10^9^/L)	16.48±1.43	14.53±1.09
Lymphocytes (10^9^/L)	10.51±1.62	8.80±0.65
Neutrophils (10^9^/L)	5.05±0.63	4.40±0.18
Platelets (10^9^/L)	230.50±23.80	229.20±16.67

Data were presented as mean±standard error. p≤0.05 was considered significant. NEFA=Non-esterified fatty acids, TAC=Total antioxidant capacity, MDA=Malondialdehyde, RBCs=Red blood cells, PCV=Packed cell volume, HB=Hemoglobin, WBCs=White blood cells

### Hematologic study

Hematologic profiles of postpartum primiparous and multiparous cows demonstrated a significant decrease in red blood cell (RBC) count and packed cell volume (PCV) but a significant increase in lymphocytes and also showed a non-significant reduction in hemoglobin when compared to those of the prepartum groups (Tables-1 and 2). When compared to the postpartum primiparous cows, the multiparous cows revealed a non-significant decrease in RBCs and hemoglobin (Table-3).

### Milk yield and BCS

A significant increase in milk yield and BCS was found in the multiparous group compared to the postpartum primiparous group (Figure-1).

## Discussion

The paper was aimed at elucidating the modifications in metabolic profile patterns of Holstein dairy cows during the transition period in both the primiparous and multiparous groups as well as assessing the consequences of parity on hematobiochemical parameters and oxidative stress biomarkers during the period of transition from pregnancy to lactation. Monitoring these alterations is highly crucial in evaluating the health status of herds as well as predicting the incidence of metabolic problems at this essential phase. Based on the study results, both the postpartum primiparous and multiparous groups showed alterations in different hematobiochemical parameters after parturition, and we observed that NEB was more evident in postpartum primiparous cows. Oxidant/antioxidant imbalance occurred in both postpartum primiparous and multiparous groups during the transition stage.

Metabolic profiling is essential in determining animal status. Parity, production season, and lactation stage are factors taken into account when assessing metabolic standpoint of dairy cows [6]. At early lactation stage, approximately 80% of blood constituents are essentially processed during milk production [12]. Hence, lactation has a strong impact on body storages and reserves in cows [13].

In this study, postpartum primiparous cows showed significant decrease in glucose, total protein, and albumin while revealing significant increase in MDA, NEFA, and globulin when compared to the prepartum primiparous group. The previous studies found higher NEFA concentrations in primiparous cows between 2 and 8 weeks postpartum [14,15]. However, another previous report has documented that NEFA concentration began to increase from 1-week prepartum until calving subsequently declining after parturition [16]. This indicates that the increase in NEFA in primiparous cows may be related to the energy demands of growth, load of the first lactation, and stressors of calving on these animals. Furthermore, studies support the fact that primiparous cows are more influenced by energy requirement deficits in the prepartum and postpartum periods [17,18]. The significant decrease of total protein during this phase occurs because ruminants utilize amino acids (from protein breakdown) as a vital fuel source for energy generation and synthesis [19].

In this study, hypoglycemia in postpartum primiparous cows was realized, which agrees with a previous study [16]. Increased demand for glucose to cope with the expected increase in milk production causes hypoglycemia during this phase [20]. In one study, glucose was thought of as an insensitive indicator of cattle energy status due to homeostatic regulation [5]; however, it was hypothesized that glucose in conjunction with NEFA and β-HBA is useful in indicating an animal’s energy status [21].

Evaluation of the multiparous cows’ metabolic profile pattern revealed significant decrease in glucose, total protein, and albumin, with significant increase in cholesterol and MDA, when compared to prepartum multiparous state. The reduction of glucose level in postpartum phase was also reported in other studies [16,20]. Increased glucose demand during early lactation, particularly in high-yielding animals, results in a hypoglycemic state [22]. NEB, associated with early lactation phase in high-yielding dairy cows, forces the animal’s body to utilize its protein and fat reserve to compensate, which explains the reduction in total protein concentration after parturition [23].

Furthermore, significant increase in cholesterol in the post-parturition phase was noted in the multiparous group. This finding agreed with results of the previous studies stating that serum cholesterol concentration is comparatively higher in multiparous cows during early lactation, which contributes to fat mobilization that takes place during this time [15,16,24,25]. In addition, cholesterol concentration at the last days of pregnancy and around calving was relatively lower when compared to the amount of concentration during the 2^nd^ week of lactation due to increased needs during fetal development, in addition to the requirement of the ovaries for synthesis of steroid hormones [15,16,24,25]. The higher increase in cholesterol levels in multiparous cows could be associated with greater tissue mobilization, increased food intake, and more synthesis of steroid hormones and lipoproteins, which are normal physiological processes of the post-parturition phase [26,27].

In addition, in previous papers, comparison between postpartum primiparous and multiparous cows revealed non-significant differences in biochemical factors, including glucose, total protein, albumin, cholesterol, and triglycerides [20,25], which confirms the results found in the present paper.

When comparing postpartum primiparous with postpartum multiparous cows, the most striking result was the significant decrease in NEFA found in the latter group. Contradictory results were reported in the previous studies; however, some researchers asserted that NEFA changes are more pronounced in multiparous cows in the postpartum phase [14,15,17]. Other reports showed no significant differences in NEFA between multiparous and primiparous cows [20,25]. Furthermore, another paper stated that NEFA is more correlated with lactation stage than with parity [6]. The pervious investigators hypothesized that negative energy imbalance is more noticeable in primiparous heifers explained in their growth requirement, fetal development, and lactation, coupled with reduced appetite after parturition [15,17,18].

Recently, an increasing interest in free-radical damage has been developed for the assessment of metabolic condition in transition cows. Under typical physiological circumstances, the body has a natural way of combating free radical build-up with the help of adequate antioxidants [8]. The MDA and TAC values in transition cows can reflect the physiological equilibrium during this phase [28]. The findings of the current study found that the level of MDA was significantly higher in both the primiparous and multiparous groups at postpartum period. The previous research studies consistently report similar findings [29-33]. MDA is produced from polyunsaturated fatty acid peroxidation and is used as an indicator for lipid peroxidation [34,35]. A significant mobilization of NEFA in dairy cows associated with increase in free oxygen radicals (e.g., ROS) production [36]. Increased free-radical production could be associated with the increase in MDA levels [37].

In the current study, TAC was selected to define the balance between pro-oxidants (MDA) and antioxidants [38]. Estimation of TAC could be used to give a rough indication of the antioxidant arm [38,39]. This study showed non-significant elevation in TAC in both postpartum primiparous and multiparous cows. Similar with a previous study, no correlation could be established between oxidant and antioxidant levels in postpartum cows [40]. Moreover, the highest level of TAC was recorded in dairy cow at the 8^th^ week of lactation [30]. This explains why production of peroxides exceeds the antioxidant system’s capacity, considering the fact that these animals are predisposed to oxidative stress status, which might lead to negative impacts on their health status [41]. Oxidative stress status can occur because of massive oxidant output and/or depletion of defenses against oxidant attacks [42]. In relation to the outcomes presented in this study, we highlight the importance of oxidative stress profile along with metabolic profile in transition cows as a complementary tool in the assessment of metabolic status.

HP has been used recently as predictive indicator for clinical metritis, ketosis, and retained placenta occurrence at postpartum phase in dairy cows [43]. In the present study, no significant variations in HP concentration between the different groups were detected. Earlier articles have noted significant increase in HP concentration in the 1^st^ week postpartum [44]. Nevertheless, a previous report agreed with our results and found a non-significant increase in HP at the post-parturient period [45]. These differences might occur due to interindividual variability in the normal physiological acute phase response to parturition as some cows did not show any increase in HP concentrations at the early post-parturient phase [43]. In addition, a previous research assumed that primiparous cows that calve spontaneously might suffer from mild cervical or vaginal trauma after calving which remains unobserved clinically; however, stimulated inflammatory response is associated with greater HP concentrations. Nevertheless, another study noticed that an increase in HP concentration was associated with acute infection and not with noninfectious conditions and that the trauma of normal parturition did not cause increase in HP concentrations, suggesting that increase in HP is properly associated with infection and not trauma [46,47].

In the present investigation, both the primiparous and multiparous cases showed reduction in erythrogram in the form of reduction in PCV and RBCs postpartum compared to prepartum status. A negative correlation between milk production and PCV was postulated [48]. Furthermore, similar findings were observed in other ruminants as well [13]. Increased RBC destruction in mammary cells is associated with reduction in hematocrit [49]. One study found that the highest hemoglobin and PCV was found in non-pregnant heifers and the lowest values were found in lactating cows [50]. Erythrogram tended to decline at the early lactation phase and increased at the mid-lactation phase as noted in one study [51].

The observed increase in lymphocytes postpartum in both primiparous and multiparous cows could be attributed to migration of these cells to milk to boost immune defense rather than the possibility of microbial invasion to mammary cells and elevate phagocytic ability [52]. Changes in total leucocyte count and differential leucocyte count could be attributed to stress with consequent release of cortisol in the blood stream, which, in turn, increases the number of leukocytes in circulation [53]. Lymphocyte decrease at the final stage of gestation is associated with production of large amounts of adrenocorticoids in both the cow and the fetus [54]. Several factors in reducing leukocyte count in late pregnancy have been identified, for example, diet, hormonal level, and managemental strategies [55]. The effect of lactation leads to a wide variation in lymphocyte count [56]. Furthermore, the amount of leukocyte and lymphocyte produced is related to age and gestation duration [57].

The BCS is an easy and effective way of assessing body energy and adipose tissue stores in milk-yielding cows [58]. In this study, milk yield and BCS were found to be significantly higher in multiparous cows, which are consistent with other previous related studies [14,15].

However, other authors have not established significant alterations in BCS between primiparous and multiparous cows at postpartum phase [17]. The fat stores in primiparous cows are lesser compared to those in multiparous cows, and their energy needs are greater due to continued growth. Consequently, energy requirements and utilization are different. Ultimately, physiological variations between primiparous and multiparous cows might influence body condition [14].

## Conclusion

Based on the findings derived in this study, metabolic and oxidative modifications occur in both primiparous and multiparous cows during the transition stage; however, primiparous cows are more prone to NEB impacts, which are revealed in the increase in NEFA, compared to their multiparous counterparts. The oxidant/antioxidant imbalance recorded for the both postpartum primiparous and multiparous cows highlights the importance of utilizing oxidative stress profiling in conjunction with standard metabolic profile tests in evaluating metabolic health status in dairy cows.

## Authors’ Contributions

NYS and SGY designed the experiment. EAM performed Physical examinations and samples collection and processing. NYS, SGY, and ESR performed samples analysis. SGY performed data collection. NYS and ESR wrote the first draft. ESR performed Statistical analysis. NYS, SGY, and ESR: contributed in writing, organization and revision of the whole paper in final form.. All authors read and approved the final manuscript.
